# Supplementary material for: Savory Signaling: T1R Umami Receptor Modulates Endoplasmic Reticulum Calcium Store Content and Release Dynamics in Airway Epithelial Cells
Source: Nutrients. 2023 Jan 18;15(3):493. doi: 10.3390/nu15030493 (PMC9919336; doi:10.3390/nu15030493)
Supplement: Supplementary file 1 [file nutrients-15-00493-s001.zip › nutrients-2135128-supplementary.pdf]

Supplementary Materials

Antibodies Used	Source	Catalogue Number
anti-T1R1	ThermoFisher Scientific	PA528771
anti-T1R3	Abcam	ab150525
**Cell Lines Used**	**Source**	**Catalogue Number**
Beas-2B	ATCC	CRL-9609
Primary human bronchial epithelial cells	Lonza	CC-2450
Primary nasal epithelial cells	This Study	N/A
**Chemicals Used**	**Source**	**Catalogue Number**
Bovine Serum Albumin	Millipore Sigma	A2153
CellEvent Caspase 3/7 Reagent	ThermoFisher Scientific	C10423
DAF-FM diacetate	ThermoFisher Scientific	D23844
Denatonium Benzoate	Millipore Sigma	D5765
Fluo-8 AM	Abcam	ab142773
Forskolin	Millipore Sigma	F3917
Fura-2 AM	ThermoFisher Scientific	F1221
Isoproterenol	Millipore Sigma	I6504
Lipofectamine 3000	ThermoFisher Scientific	L3000075
MEM Amino Acids	ThermoFisher Scientific	11130051
Non-Essential Amino Acids	ThermoFisher Scientific	11140050
Thapsigargin	Cayman Chemical	10522
UTP	ThermoFisher Scientific	AAJ23160-03
**Recombinant DNA - Function**	**Source**	**Catalogue Number**
AKAR4 - intracellular PKA activity	Addgene	61619
AKAR4-nls - nuclear PKA activity	Addgene	138217
Flamindo2 - intracellular cAMP	Addgene	73938
nls-Flamindo2 - nuclear cAMP	Addgene	73939
**RNAi used**	**Source**	**Catalogue Number**
TAS1R1 DsiRNA Kit	Integrated DNA Technologies	hs.Ri.TAS1R1.13
TAS1R3 DsiRNA Kit	Integrated DNA Technologies	hs.Ri.TAS1R3.13
**Taqman Probes for qPCR**	**Source**	**Catalogue Number**
GAPDH	ThermoFisher Scientific	Hs02786624_g1
TAS1R1	ThermoFisher Scientific	Hs01547926_g1
TAS1R2	ThermoFisher Scientific	Hs00541095_m1
TAS1R3	ThermoFisher Scientific	Hs00877446_g1
TAS2R4	ThermoFisher Scientific	Hs00249946_s1
TAS2R10	ThermoFisher Scientific	Hs00256794_s1
TAS2R14	ThermoFisher Scientific	Hs00256800_s1
TAS2R16	ThermoFisher Scientific	Hs00249955_s1
TAS2R38	ThermoFisher Scientific	Hs00604294_s1
TAS2R40	ThermoFisher Scientific	Hs00602589_s1
TAS2R43	ThermoFisher Scientific	Hs00853105_sH
TLR4	ThermoFisher Scientific	Hs00152939_m1
TLR5	ThermoFisher Scientific	Hs01920773_s1
UBC	ThermoFisher Scientific	Hs01871556_s1

Table S1. A list of reagents used in this study.

a

MEM Amino Acids, Essential Amino Acids

ThermoFisher Product Number 11130051

Amino Acid	1x Concentration (mM)
L-Arginine hydrochloride	0.60
L-Cystine	0.10
L-Histidine hydrochloride-H2O	0.20
L-Isoleucine	0.40
L-Leucine	0.40
L-Lysine hydrochloride	0.40
L-Methionine	0.10
L-Phenylalanine	0.20
L-Threonine	0.40
L-Tryptophan	0.05
L-Tyrosine	0.20
L-Valine	0.40

b

MEM Non-Essential Amino Acids

ThermoFisher Product Number 11140050

Amino Acid	1x Concentration (mM)
Glycine	0.1
L-Alanine	0.1
L-Asparagine	0.1
L-Aspartic acid	0.1
L-Glutamic Acid	0.1
L-Proline	0.1
L-Serine	0.1

Table S2. Formulations of 1x MEM AA (a) and 1x NEAA (b) used in this study.

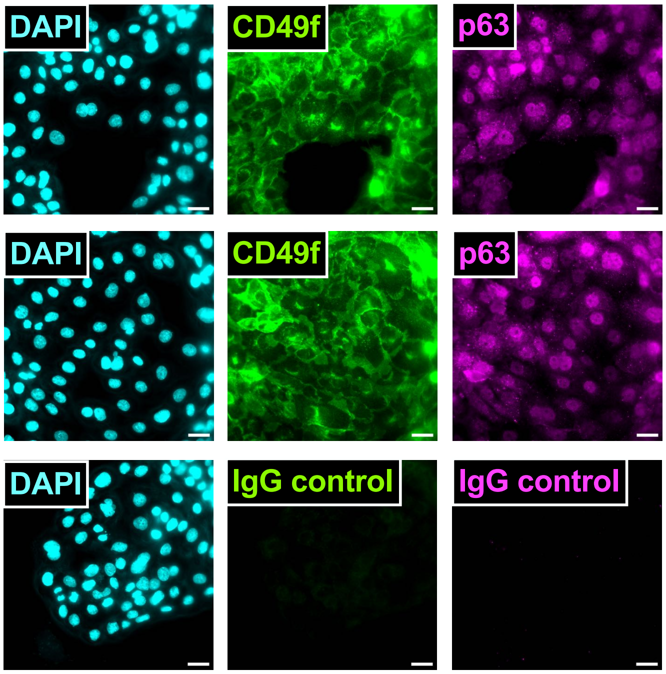

Figure S1. Primary nasal epithelial cells cultured in Pneumacult express basal cell markers CD49f and p63. PneumaCult-Ex Plus media enhances basal cell phenotype and increases expression of basal cell markers like CD271 and CD49f (StemCell Technologies technical document #27188, "PneumaCultTM Culture Media for Human Airway Epithelial Cells" https://cdn.stemcell.com/media/files/brochure/BR27188-PneumaCult_Culture_Media_for_Human_Airway_Epithelial_Cells.pdf accessed Jan 9, 2023). We confirmed this by performing immunofluorescence staining for two airway epithelial basal cell markers: CD49f (α6 integrin; BD 561894; rat primary antibody) [1-4] and the p63 transcription factor (Cat # AF1916, R&D Systems; goat primary antibody) [5-7]. Immuno-fluorescence co-staining revealed expression and proper-appearing localization of both CD49f and p63 (membrane vs nucleus, respectively), confirming the basal cell nature of the cells. Cultures of primary basal cells grown on glass bottom dishes (MatTek) were fixed in 4% paraformaldehyde for 15 min at room temperature then permeabilized and blocked using phosphate buffered solution containing 0.1% Triton X-100, 5% normal donkey serum, 0.2% saponin for 45 min at room temperature. Primary antibody was diluted 1:100 in the blocking buffer (without Triton X-100) and incubated overnight. Cultures were then incubated in secondary anti-rat or anti-goat AlexaFluor-labeled antibodies (ThermoFisher Scientific) for 1 hour at 4°C then mounted with Fluoroshield with DAPI (Abcam). All images were taken on an Olympus IX-83 microscope with a 60x objective (1.4 NA PlanApo oil) using MetaMorph and analyzed via ImageJ/FIJI [8]. Rat IgG and Goat IgG plus secondary antibodies were used as controls for non-specific staining (bottom row, taken at identical microscope settings with min/max levels adjusted identically to rows above). Results are representative of cells from n = 3 patients examined for this study. Scale bar is 20 µm.

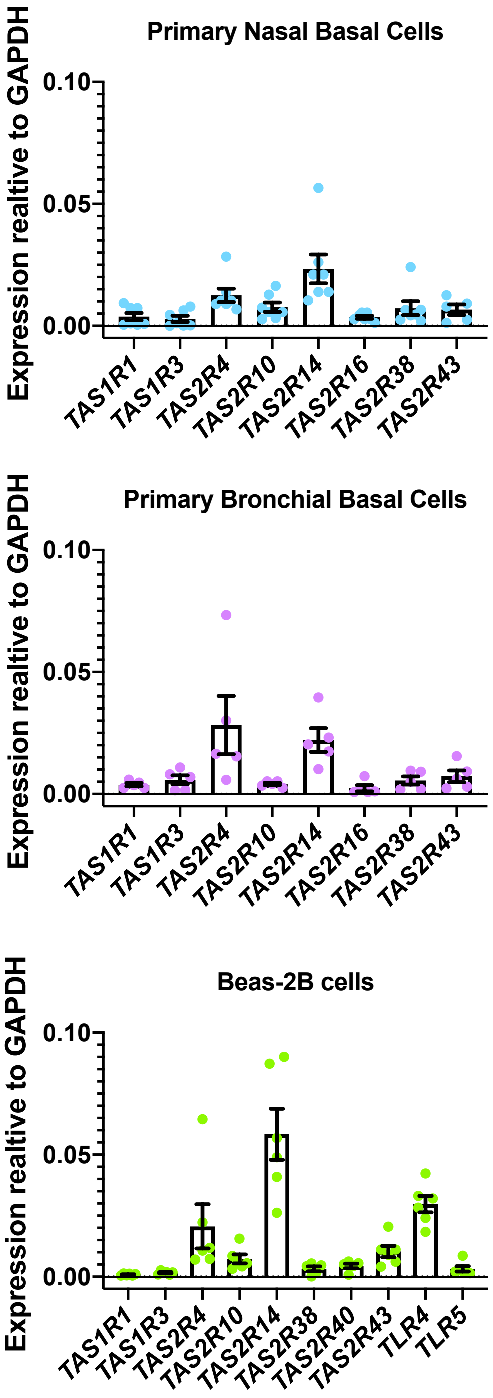

Figure S2. Relative expression (normalized to GAPDH) of TAS1R and TAS2R genes in primary nasal basal cells (left), primary bronchial basal cells (middle) and Beas2B cells (right). TLRs 4 and 5 are also shown for Beas-2B cells. Note that while TLR5 expression is lower than TLR4, we previously showed that TLR5 is functional in Beas2B cells and responds to *Pseudomonas aeruginosa* flagellin [9]. Taqman probes used are listed in Supplementary Table S1.

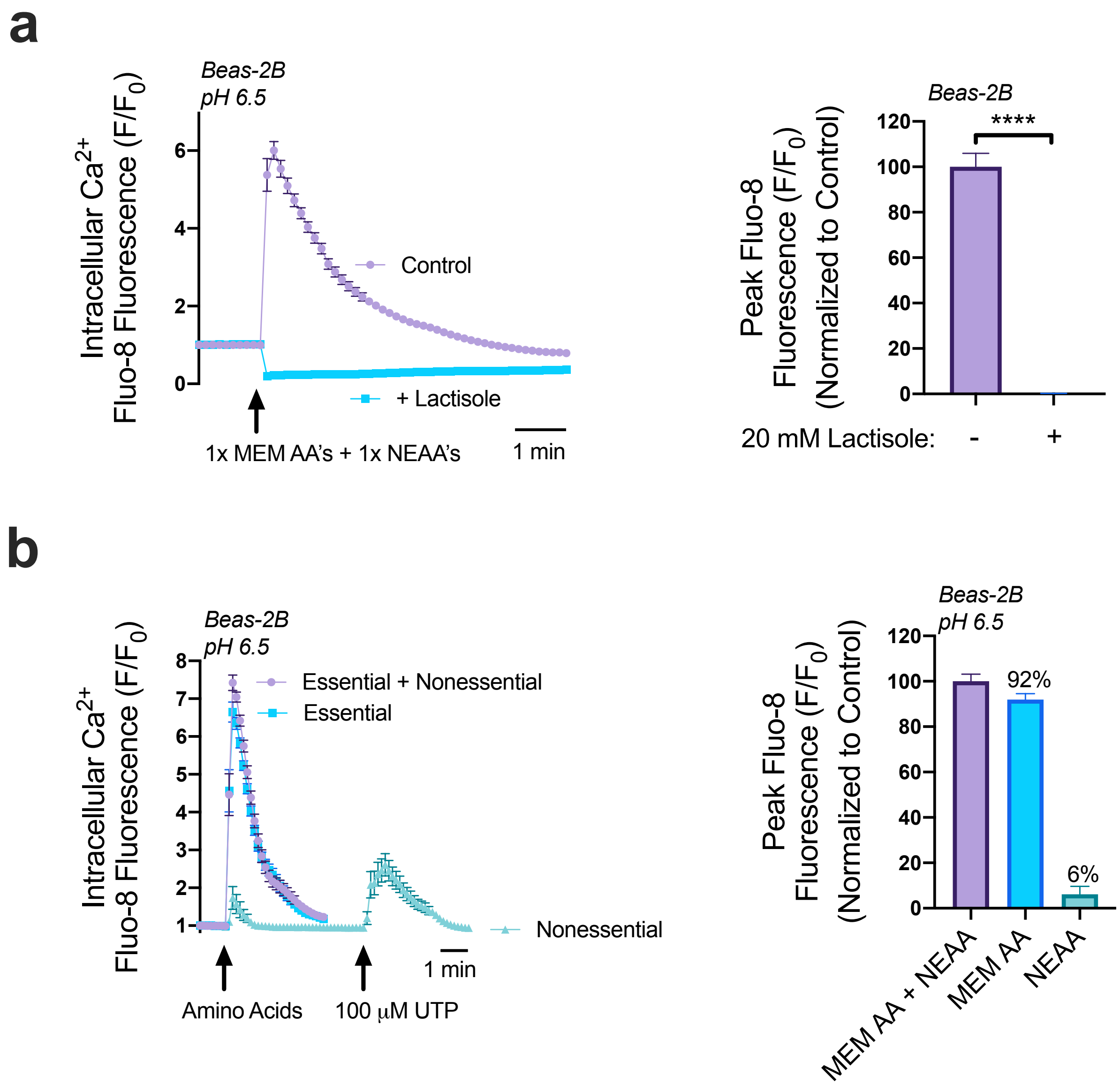

Figure S3. Amino acids induce Ca^{2+} elevations in Beas-2B's at pH 6.5. (a) Beas-2B's were loaded with Ca^{2+} detecting dye Fluo-8 AM and pre-treated with 20 mM lactisole for 1 hour then stimulated with a mixture of 1x MEM AA and 1x NEAA's. At pH 6.5 1x MEM AA's and 1x NEAA stimulate a Ca^{2+} response that is inhibited by lactisole. (b) Maintaining a pH of 6.5, the majority of the Ca^{2+} elevations (approximately 92%) are due to components of the 1x MEM AA mixture while 1x NEAA contributed minimally to Ca^{2+} signaling pathways (approximately 6% of the combined release). Significance determined by Student's t-test ****P < 0.0001

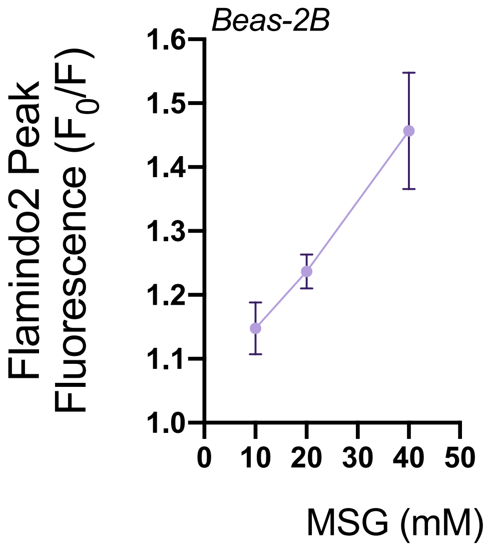

Figure S4. Stimulation with monosodium glutamate (MSG) dose-dependently increased Flamindo2 F_0/F, indicating cAMP increases. Note that this is within the range of MSG concentrations that have been shown to activate T1R1/3 in the absence of IMP [10-12].

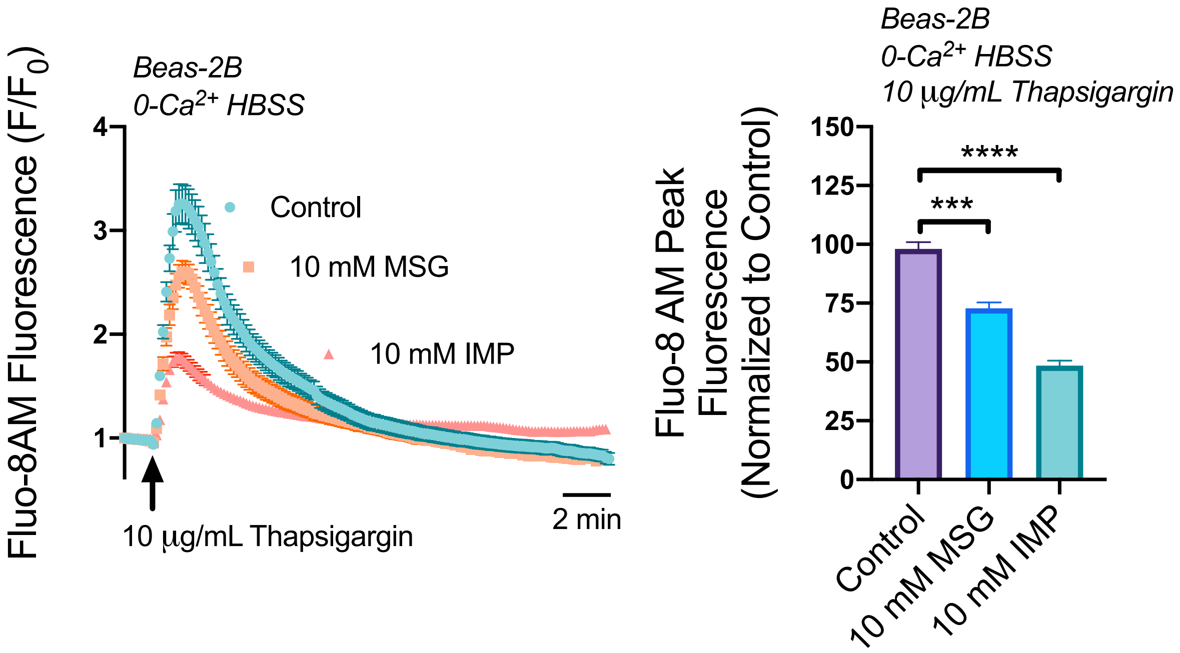

Figure S5. Pre-treatment with umami agonists 10 mM monosodium glutamate (MSG) or 10 mM inosine monophosphate (IMP) reduces ER Ca^{2+} content similarly to MEM AA's shown in the main text. Pretreatment and thapsigargin stimulation was performed identically as with MEM AA's in the main text. Representative traces shown on left and bar graph of 3-5 independent experiments shown on right. Significance by one-way ANOVA with Dunnett's posttest comparing values to control (no pre-treatment); ****P*< 0.001, *****P* < 0.0001.

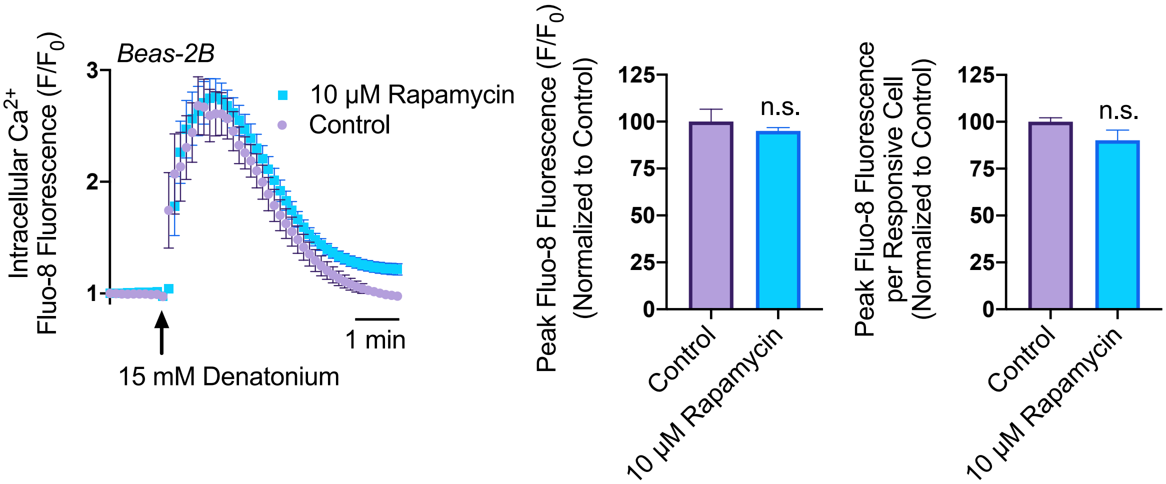

Figure S6. mTORC1 pathways do not impact ER Ca^{2+} content. Beas-2B's loaded with Fluo-8 AM were treated with 10 µM of rapamycin for 1 hour then stimulated with 15 mM denatonium. Rapamycin had no impact on the population's peak Ca^{2+} levels or the peak Ca^{2+} release per cell, showing that there was no observable effect on Ca^{2+} signaling pathways. Significance determined by Student's *t*-test 'n.s.' represents no significance.

Supplementary References

1. Li, F.; He, J.; Wei, J.; Cho, W.C.; Liu, X. Diversity of epithelial stem cell types in adult lung. *Stem Cells Int* **2015**, *2015*, 728307, doi:10.1155/2015/728307.
2. Ghosh, M.; Ahmad, S.; Jian, A.; Li, B.; Smith, R.W.; Helm, K.M.; Seibold, M.A.; Groshong, S.D.; White, C.W.; Reynolds, S.D. Human tracheobronchial basal cells. Normal versus remodeling/repairing phenotypes in vivo and in vitro. *Am J Respir Cell Mol Biol* **2013**, *49*, 1127-1134, doi:10.1165/rcmb.2013-0049OC.
3. Bonser, L.R.; Koh, K.D.; Johansson, K.; Choksi, S.P.; Cheng, D.; Liu, L.; Sun, D.I.; Zlock, L.T.; Eckalbar, W.L.; Finkbeiner, W.E., et al. Flow-Cytometric Analysis and Purification of Airway Epithelial-Cell Subsets. *Am J Respir Cell Mol Biol* **2021**, *64*, 308-317, doi:10.1165/rcmb.2020-0149MA.
4. Rock, J.R.; Onaitis, M.W.; Rawlins, E.L.; Lu, Y.; Clark, C.P.; Xue, Y.; Randell, S.H.; Hogan, B.L. Basal cells as stem cells of the mouse trachea and human airway epithelium. *Proc Natl Acad Sci U S A* **2009**, *106*, 12771-12775, doi:10.1073/pnas.0906850106.
5. Bilodeau, C.; Shojaie, S.; Goltsis, O.; Wang, J.; Luo, D.; Ackerley, C.; I, M.R.; Cox, B.; Post, M. TP63 basal cells are indispensable during endoderm differentiation into proximal airway cells on acellular lung scaffolds. *NPJ Regen Med* **2021**, *6*, 12, doi:10.1038/s41536-021-00124-4.
6. Di Como, C.J.; Urist, M.J.; Babayan, I.; Drobnjak, M.; Hedvat, C.V.; Teruya-Feldstein, J.; Pohar, K.; Hoos, A.; Cordon-Cardo, C. p63 expression profiles in human normal and tumor tissues. *Clin Cancer Res* **2002**, *8*, 494-501.
7. Chilosi, M.; Doglioni, C. Constitutive p63 expression in airway basal cells. A molecular target in diffuse lung diseases. *Sarcoidosis Vasc Diffuse Lung Dis* **2001**, *18*, 23-26.
8. Schindelin, J.; Arganda-Carreras, I.; Frise, E.; Kaynig, V.; Longair, M.; Pietzsch, T.; Preibisch, S.; Rueden, C.; Saalfeld, S.; Schmid, B., et al. Fiji: an open-source platform for biological-image analysis. *Nat Methods* **2012**, *9*, 676-682, doi:10.1038/nmeth.2019.
9. Kuek, L.E.; McMahon, D.B.; Ma, R.Z.; Miller, Z.A.; Jolivert, J.F.; Adappa, N.D.; Palmer, J.N.; Lee, R.J. Cilia Stimulatory and Antibacterial Activities of T2R Bitter Taste Receptor Agonist Diphenhydramine: Insights into Repurposing Bitter Drugs for Nasal Infections. *Pharmaceuticals (Basel)* **2022**, *15*, doi:10.3390/ph15040452.
10. Zhao, G.Q.; Zhang, Y.; Hoon, M.A.; Chandrashekar, J.; Erlenbach, I.; Ryba, N.J.; Zuker, C.S. The receptors for mammalian sweet and umami taste. *Cell* **2003**, *115*, 255-266.
11. Ozeck, M.; Brust, P.; Xu, H.; Servant, G. Receptors for bitter, sweet and umami taste couple to inhibitory G protein signaling pathways. *Eur J Pharmacol* **2004**, *489*, 139-149, doi:10.1016/j.ejphar.2004.03.004.
12. Li, X.; Staszewski, L.; Xu, H.; Durick, K.; Zoller, M.; Adler, E. Human receptors for sweet and umami taste. *Proc Natl Acad Sci U S A* **2002**, *99*, 4692-4696, doi:10.1073/pnas.072090199072090199 [pii].
